# PPARs as Nuclear Receptors for Nutrient and Energy Metabolism

**DOI:** 10.3390/molecules24142545

**Published:** 2019-07-12

**Authors:** Fan Hong, Shijia Pan, Yuan Guo, Pengfei Xu, Yonggong Zhai

**Affiliations:** 1 Beijing Key Laboratory of Gene Resource and Molecular Development, College of Life Sciences, Beijing Normal University, Beijing 100875, China; 2 Key Laboratory for Cell Proliferation and Regulation Biology of State Education Ministry, College of Life Sciences, Beijing Normal University, Beijing 100875, China; 3 Center for Pharmacogenetics and Department of Pharmaceutical Sciences, University of Pittsburgh, Pittsburgh, PA 15213, USA

**Keywords:** PPARs, nutrition, energy metabolism, selective agonist

## Abstract

It has been more than 36 years since peroxisome proliferator-activated receptors (PPARs) were first recognized as enhancers of peroxisome proliferation. Consequently, many studies in different fields have illustrated that PPARs are nuclear receptors that participate in nutrient and energy metabolism and regulate cellular and whole-body energy homeostasis during lipid and carbohydrate metabolism, cell growth, cancer development, and so on. With increasing challenges to human health, PPARs have attracted much attention for their ability to ameliorate metabolic syndromes. In our previous studies, we found that the complex functions of PPARs may be used as future targets in obesity and atherosclerosis treatments. Here, we review three types of PPARs that play overlapping but distinct roles in nutrient and energy metabolism during different metabolic states and in different organs. Furthermore, research has emerged showing that PPARs also play many other roles in inflammation, central nervous system-related diseases, and cancer. Increasingly, drug development has been based on the use of several selective PPARs as modulators to diminish the adverse effects of the PPAR agonists previously used in clinical practice. In conclusion, the complex roles of PPARs in metabolic networks keep these factors in the forefront of research because it is hoped that they will have potential therapeutic effects in future applications.

## 1. Introduction

It is well known that chemicals that cause peroxisome proliferators can specifically act on a variety of tissues and cells through specific mediators. When mice were given drugs (such as nafenopin) that reduced plasma triglyceride levels, the activity of long-chain fatty acids and catalase oxidase [1,2,3] was observed to increase with the proliferation of peroxisomes in liver parenchymal cells [4]. To explore the mechanism, in 1983 ND Lalwani et al. found a cytoplasmic protein that can reversibly and specifically bind to nafenopin in rat liver in a manner related to the induction of peroxisome proliferation by hypolipidemic compounds. Therefore, it was speculated that peroxisomal proliferation can be mediated by receptors [5]. In 1987, ND Lalwani purified a dimeric protein from rat hepatocytes, which binds to a peroxisome proliferator [6]. Further studies have shown that this binding protein is homologous to the heat shock protein 70 (HSP70), but how it mediates peroxisome proliferation is unclear [7]. In 1990, two researchers from Central Toxicology Laboratory discovered a new ligand-activated transcription factor from mouse cDNA libraries that was determined to be a new member of the steroid hormone receptor superfamily [8]. The receptor is structurally related to the steroid hormone receptor, but the two receptors are significantly different. The newly discovered protein can be activated by various molecules, such as fatty acids or fibrates, and mediates the peroxisome proliferative response by a specific receptor called PPAR, which was subsequently named PPARα (NR1C1) [8]. It is well known that this receptor was discovered in murine animals [8], and was subsequently found in other species, such as frog [9], rat [10], human [11], and rabbit [12], and that it was a target for hypolipidemic fibrates [8]. The first fibrates were synthesized in the mid-1950s and, later, other fibrates were developed in Europe, such as bezafibrate and ciprofibrate, which are a widely used class of hypolipidemic drugs. In the early 1990s, it was demonstrated by gene knockout mice that the hypolipidemic effect of fibrates was regulated by PPARα. Fibrates reduce plasma lipid concentration and induce hepatic swelling and peroxisome proliferation have been shown to act in a PPARα-dependent manner in PPARα knockout mice [9].

**Figure 1:**
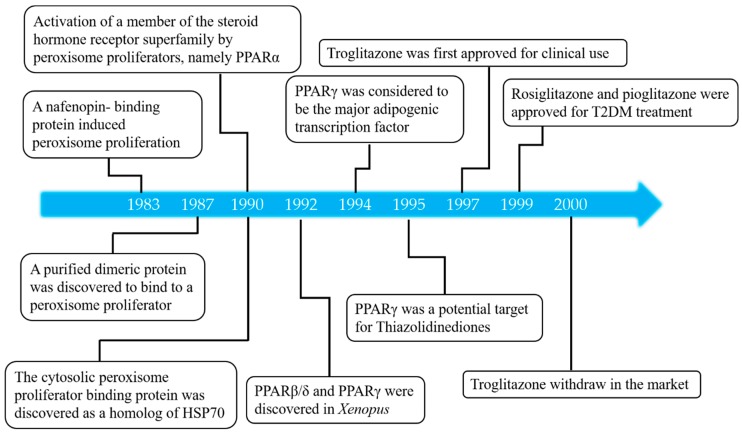
Discovery of the PPARs. Landmark events in the advancement of PPARs research.

Two genes, namely PPARβ/δ (NR1C2) and PPARγ (NR1C3), from the same family of PPARα, were subsequently cloned in 1992 after the discovery of PPARα [9]. Human [13] and *Xenopus* [9] PPARβ/δ was discovered in 1992 and subsequently cloned from mice [14] and rats [15]. PPARβ/δ is activated by some saturated and polyunsaturated fatty acids or eicosanoids. Synthetic PPARβ/δ ligands have been developed, such as GW501516, but have not yet been approved for clinical treatment. Then, PPARγ was discovered in *Xenopus* in 1992 [9] and subsequently cloned from mice [16] and human [17]. In 1994, PPARγ was considered to be the major adipogenic transcription factor [18]. Thiazolidinediones (TZDs), a class of drugs that are derivatives of thiazolidinedione, are selective activators of PPARγ and are widely used to treat type 2 diabetes (T2DM). BRL49653 is the most potent agent and can induce the differentiation of pluripotent C3H10T1/2 stem cells into adipocytes; thus, PPARγ was shown to be a potential target for TZD in 1995 [19]. Among the discovered PPAR activators, in 1997, troglitazone was the first applied to clinical use because it promotes insulin sensitivity by increasing glucose utilization and decreasing glucose production. However, it was pulled from the market in 2000 because it induces serious liver toxicity. Subsequently, rosiglitazone and pioglitazone were approved for the clinical treatment of T2DM in 1999 and they are the only two TZDs that have been used to treat this disease to date. As they are closely related to obesity and cardiovascular disease, these receptors have been widely studied since their identification [20,21].The mainly landmark events in the advancement of PPARs research are shown in Figure 1.

PPARs are activated by different ligands and participate in different physiological responses, such as metabolism and energy homeostasis [22,23]. The three PPAR subtypes are highly homologous but are distributed in different tissues, encoded by different genes [24], and show different distribution patterns within tissues and biological functions. PPARα is mainly expressed in liver, brown adipose tissue (BAT), and heart, kidney, and muscle tissue [20], and it is mainly involved in β-oxidation and fatty acid transport to regulate lipid balance [25]. PPARβ/δ is universally expressed in skeletal muscle, adipose tissue, the heart, and the gastrointestinal tract, and it is mainly involved in fatty acid metabolism. PPARγ is expressed in adipose tissue, immune cells, and the colon, and it is mainly responsible for regulating adipocyte differentiation and improving insulin resistance [20,25]. As nuclear receptors, PPARs are widely distributed in different organs, so their roles in nutrient and energy metabolism need to be further explored.

In this review, we highlight the role of various PPAR isoforms in several major organs and describe their mechanisms for maintaining energy homeostasis and body health to better inform future drug development.

## 2. Action of PPARs in Nutrient Metabolism

As nutrient sensors, PPARs balance nutrient metabolism and maintain the metabolic flexibility involved in lipid metabolism, glucose homeostasis, cholesterol metabolism, and other significant metabolic networks. The endogenous agonists of PPARs are a class of fatty acids and their derivatives that are mainly produced in one of three ways, as follows: Diet, de novo lipogenesis, and lipolysis [26]. For example, dietary lipids, which usually have unsaturated fatty acids, can activate PPARs (all three types), phospholipids (PPARα in particular), 15d-PGJ2 (PPARγ in particular), prostacyclin I2 (PPARβ/δ in particular), and so on [25]. In addition, the synthetic ligands of PPARs have also contributed to nutrient metabolism control, especially in diet-induced metabolic disorders, since the initiation of drug research. There are a series of PPAR agonists, such as fibrate, TZDs, and GW501516, that enhance the power of PPARs in regulating lipid and glucose metabolism during different nutritional states.

### 2.1. PPARs in Lipid Metabolism

In the fasting state, PPARα accelerates fatty acid formation caused by lipolysis influx in adipose tissue of the liver by regulating the expression of apolipoprotein, thus increasing plasma levels of high-density lipoprotein cholesterol (HDL-C) and reducing levels of low-density lipoprotein cholesterol (LDL-C) [27,28,29,30,31,32]. Promoting mitochondrial or peroxisomal oxidation in the liver is also vital for PPARα action in protecting the liver from lipotoxicity [33]. In addition, during long-term starvation, PPARα also induces ketone body production and then uses it for energy supply in extrahepatic tissue [34]. Activated PPARγ can decrease free fatty acid content in all organs except adipose tissue and circulating blood, consequently improving the capacity of adipose tissue to store triglyceride (TG) [25]. However, this effect of PPARγ was blunted in white adipose tissue (WAT) during fasting, and its mechanism involved activating sirtuin 1 (SIRT1) [35] or AMP-activated protein kinase (AMPK) [36].

During a state of adequate nutrition, PPARα plays another role. It coordinates de novo lipogenesis to produce fatty acids, which store the energy reserves for use during starvation [34]. In contrast to PPARα, PPARγ is usually activated in the fed state, which facilitates fatty acid transport to WAT and then improves lipid synthesis and storage [34].

There is no distinct difference in the role of PPARβ/δ in different nutritional states. However, it synergistically improves fatty acid catabolism in skeletal muscle and inhibits lipogenesis in adipose tissue [37]. It has been reported that PPARβ/δ decreases the stability of sterol regulatory element-binding protein 1C (SREBP1C), which upregulates lipogenesis by activating Insig-1 and thus prevents lipid accumulation in the liver [38,39]. Moreover, PPARβ/δ also elevates the thermogenesis function of BAT by upregulating the transcription of specific genes, including uncoupling protein 1 (UCP1) and fatty acid oxidation (FAO) [20].

From a global biological systems point of view, the regulation of lipid metabolism networks by PPARs is significantly important and extremely complex. The functions of PPARs in lipid metabolism remain somewhat unknown.

### 2.2. PPARs in Glucose Homeostasis

PPARα plays an antagonistic role in glucose homeostasis compared to insulin, which promotes glycolysis and de novo fatty acid synthesis but decreases gluconeogenesis. Therefore, PPARα inhibits lipid accumulation by decreasing glycolysis and improving glycogen synthesis and FAO [40]. These effects of PPARα were specifically observed in the overexpression of PPARα in mouse skeletal muscle (SKM), which showed increasing levels of glucose and insulin in plasma when mice were fed a chow diet [41]. However, the adverse effect of fibrates is mild and acceptable in clinical practice.

Interestingly, contrasting metabolic effects appeared between ligand-activated PPARα and PPARγ. PPARγ plays a vital role in glucose homeostasis, including in the enhancement of SKM sensitization to insulin, improving glucose-stimulated insulin secretion in pancreatic β-cells and increasing gluconeogenesis in the liver [42]. This effect of PPARγ is partly caused by regulation of a series of transcription proteins, such as c-Cbl-associated protein (CAP) and glucose transporter type 4 (Glut4) [42]. Another way to improve insulin signaling by PPARγ is to transfer lipids out of circulation, liver, and SKM and into WAT, which may cause adipogenesis in the WAT [34]. In addition, PPARγ induces WAT-secreting adipokines, such as adiponectin (facilitating hepatic glucose output) and leptin (regulating feeding behavior), to improve insulin sensitivity [42].

PPARβ/δ plays a major role in improving glycolysis, glucose uptake, and glycogen storage and in decreasing gluconeogenesis [43,44]. On the one hand, it boosts the conversion of type II fast-twitch glycolytic SKM to the fiber type I slow-twitch oxidative fibers by a PPARβ/δ agonist. These changes involve estrogen-related receptor γ (ERRγ)/microRNA and PGC1α pathway control, which elevates the capacity of SKM utilization of glucose [45,46]. Another way to improve insulin sensitivity by PPARβ/δ is to strengthen the pentose phosphate pathway, which improves glucose utilization and inhibits glucose efflux from the liver [43]. The mechanism of PPARs in mediating glucose homeostasis is tissue-dependent.

### 2.3. PPARs in Cholesterol Metabolism

PPARα also plays a vital role in regulating cholesterol and bile acid metabolism. In the majority of trials, PPARα activated by fibrates consistently displayed beneficial effects on decreased “atherogenic lipids,” including triglycerides and LDL-C, as well as elevated HDL-C levels in plasma [47,48,49,50,51]. Ciprofibrate, an agonist of PPARα, inhibits the expression of CYP7a1, which is a key enzyme of bile acid production in the liver [52]. Hepatic Na^+^-taurocholate cotransporting polypeptide (NTCP), organic-anion-transporting polypeptide (OATP1), and bile salt export pump (BSEP) are involved in the regulation of bile acid influx in the liver by activating PPARα, and this phenotype is abolished in PPARα-null mice [53]. Then, PPARα mediates the transport of cholesterol by enhancing apolipoprotein AI (Apo-AI) expression.

It has also been found that agonists of PPARα and PPARγ promote the expression of liver-x-receptor (LXR), which regulates ABCA1 expression, which increases the production of Apo-AI rich-HDL and induces cholesterol efflux from macrophages [54]. However, not all TZDs have the same protective effects during cardiovascular events in patients with T2DM. In the PROspective pioglitAzone Clinical Trial, pioglitazone, targeting both PPARα and PPARγ, has more favorable effects than rosiglitazone on controlling cholesterol metabolism [54]. In our previous study, Danshensu Bingpian Zhi (DBZ), a potential PPARγ agonist [55] prevented atherosclerosis by modulating LXR and inhibited inflammation, macrophage migration, and foam cells formation in ApoE^−/−^ mice fed a high cholesterol diet [56,57].

PPARβ/δ agonists appear to have similar effect as PPARα and PPARγ agonists by elevating plasma levels of HDL and declining levels of LDL. These positive effects have been validated in different rodent and primate models [58,59,60,61]. Moreover, it has been proven that PPARβ/δ also inhibits the expression of Niemann–Pick C1-like 1 (NPC1L1) in the intestine, reduces cholesterol adsorption, and improves transintestinal cholesterol efflux [62]. Regulating cholesterol metabolism is another contribution of PPARs and, in combination with other nuclear receptors such as LXR, PPARs are important for balancing homeostasis of cholesterol metabolism.

### 2.4. PPARs in Animals Fed a High-Fat Diet

In over nutrient conditions, especially in high-fat diets (HFD), PPARs have also displayed various roles in past studies. The expression of PPARα is generally decreased in HFD-induced obesity [63]. For example, the accumulation of adipose tissue in PPARα-null mice fed a HFD is more obvious and the glucose utilization level is also increased [64]. It is well known that excess fat intake or a long-term western diet can induce insulin resistance. However, this phenotype was not observed in the PPARα-null mice. In addition, activation of PPARα through fenofibrate stimulates the transcription of thermogenesis genes including UCP, PGC1α, the PR domain containing 16 (PRDM16), and fibronectin type III domain-containing protein 5 (FNDC5) in the subcutaneous WAT of mice on a HFD [65].

PPARγ have been more widely studied in recent years and many regulations of it in HFDs have been discovered. In a mouse model of HFD-induced insulin resistance, elevating PPARγ acetylation reduced the browning of WAT via inhibiting recruitment of SIRT1 to PPARγ [66,67,68]. Several studies show that the oscillating gene came to vast changes of expression in diet-induced obesity and enhancing the rhythmical expression of PPARγ could be the key to regulating a series of reprogrammed transcriptions [34,69,70]. Furthermore, Julie Tomas et al. reported that only 30 days of HFD treatment disrupted the distribution of microbiota in the intervillous zone of the ileum. However, the effect induced by a HFD was reversed after treating mice with rosiglitazone for 1 week or changing their feed to a standard diet [71]. In our previous study, DBZ inhibited HFD-induced obesity in mice by selectively activating PPARγ to a significant level and PPARα to a moderate level, but it did not activate PPARβ/δ. Conversely, several studies have reported that rosiglitazone promoted HFD-induced liver steatosis in mice, and mice with low hepatic PPARγ expression had a low incidence rate of fatty liver [72]. Interestingly, some other studies have found that both PPARα and PPARγ were increased significantly in HFD-fed mice. In addition, the offspring of these mice were fatter than those of the control group at weaning and their expression of PPARγ was increased to a level similar to that of the parents [73].

In the past decade, activated PPARβ/δ was considered to have no effect on weight loss in the treatment of obesity, but it deceased plasma levels of TG, LDL cholesterol and free fatty acids, and dyslipidemia [58]. However, it was first reported that GW501516, a PPARβ/δ agonist, protected mice on a HFD from obesity by activating the lipin1-PGC1α-PPARα pathway, thus increasing fatty acid intake and oxidation [74]. In addition, given the high expression of PPARβ/δ in the intestine, HFD- and agonist-activating PPARβ/δ promoted organoid to product progenitors and even facilitated tumor formation in vivo to inhibit the tumor suppressor adenomatous polyposis coli (APC) protein [75,76]. For mice fed a HFD, the expression of PPARs depended on many factors, including species and tissue type and period of development.

## 3. Roles of PPARs in the Energy Metabolism of Various Organs

**Figure 2:**
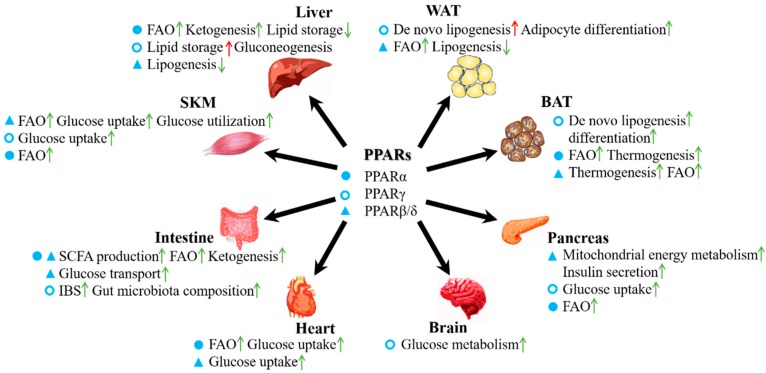
Roles of PPARs in the energy metabolism of various organs. The three types of PPARs are widely expressed in various organs, including the liver, WAT, BAT, pancreas, heart, intestine, and SKM. Regulation differences of these PPARs in different tissues are shown and the first item in each list represents the main PPAR subtype and function for the organ. The filled circle represents PPARα; the empty circle represents PPARγ; the triangle represents PPARβ/δ; the green arrow represents beneficial effect; and the red arrow represents adverse effect. For example, among the three types of PPARs, PPARα is the master regulator in the liver and the activation of it increases FAO, induces ketogenesis, and decreases lipid storage in the liver.

Although all three PPAR subtypes participate in the regulation of nutrient and energy metabolism in the body, they are distributed in different tissues and show different distribution patterns within tissues. In this section, we summarize the various roles of the three PPARs according to metabolic pathway (Figure 2).

### 3.1. PPARs in Adipose Tissue

There are two essential types of adipose tissue, WAT and BAT. Both are important endocrine organs that secrete different adipokines for systemic energy metabolism [77,78]. As is well known, among the three PPAR subtypes, PPARγ plays a dominant role in adipose tissue. It not only participates in fatty acid uptake and storage but also regulates adipose tissue differentiation [79]. Knocking out PPARγ in embryonic fibroblast inhibits their differentiation into adipocytes [80]. In the 3T3-L1 preadipocyte model, PPARγ is an important transcription factor regulating lipid accumulation, and the synthetic agonist rosiglitazone activates PPARγ to induce lipid droplet formation [81]. In laboratory research, we found that the traditional Chinese herb *Sibiraea angustata* (SA) can inhibit cell differentiation and lipogenesis by regulating the expression of PPARγ and other genes in 3T3-L1 preadipocytes to manage obesity [82]. Activation of PPARγ causes fatty acids to be transported and stored and facilitates de novo adipogenesis in adipose tissue; therefore, the PPARγ activator TZD has been generally used to treat T2DM [83]. PPARα is highly expressed in BAT but not in WAT. Under cold exposure conditions, PPAR regulates lipid oxidation and thermogenesis by interacting with PGC1α in response to β-adrenergic stimulation in BAT [84]. PPARβ/δ is also expressed in both types of adipose tissues. It controls fatty acid oxidation [85] and BAT thermogenesis to promote the expression of UCP1 and thus utilizes WAT [86]. The role of PPARβ/δ in WAT needs to be further studied [87]. In summary, PPARs may be used as drug targets for the development of safe and effective pharmaceuticals used in the treatment of obesity.

### 3.2. PPARs in the Liver

The liver is a main mediator in the metabolism of the fatty acids and glucose involved in systemic energy metabolism. PPARs and their coregulators are primary modulators of these physiological responses [88]. The expression of PPARα is ubiquitous, but is highest in the liver and plays a role in fatty acid metabolism, including mitochondrial and peroxisome fatty acid oxidation and phospholipid remodeling [89,90]. It has been reported that PPARα can affect the development of nonalcoholic fatty liver disease (NAFLD) and nonalcoholic steatohepatitis (NASH) [91,92]. Hepatocyte-restricted PPARα deletion can induce steatosis in hepatocyte-specific PPARα-null mice [93]. In conclusion, PPARα is a potential drug target in the treatment of NAFLD [93]. It is well known that PPARγ expression is lower in healthy liver [94]. In HFD-fed mice, the loss of hepatocyte-specific PPARγ results in reduced liver fat content [95]. When PPARγ is overexpressed in the liver of mice, it causes hepatic steatosis. In fatty liver, CD36, together with LXR, pregnane X receptor (PXR), and PPARγ, is involved in the regulation of free fatty acid uptake and steatosis. Whether PXR, LXR, and PPARγ interact during steatosis in lipid-related metabolite diseases such as obesity and diabetes remains to be determined [96]. PPARβ/δ is widely expressed in hepatocytes, sinusoidal endothelial cells (LSECs), and liver macrophages (Kupffer cells) [97]. The gene expression associated with the pathways of lipoprotein metabolism and glucose utilization is reduced in the liver of PPARβ/δ-null mice, indicating that the expression of these genes is positively correlated with PPARβ/δ to regulate TG and cholesterol levels [98]. All PPARs are potential drug development targets for NAFLD and NASH.

### 3.3. PPARs in the Intestine

There are a large number of microorganisms, including bacteria, viruses, and fungi, in the gastrointestinal tract in rodents and humans. Abnormal regulation of the gut microbiota leads to disorders in the internal environment, such as on the progression of atherosclerosis or the development of fatty liver. A variety of complex factors are involved in these pathological processes, of which PPARs also play a regulatory role to some extent. Importantly, PPARs play an important regulatory role in maintaining the homeostasis of the host during the interaction of different organs and intestinal microbiota [99]. PPARα and PPARβ/δ are both widely expressed in the gut. In the cecum and distal colon, they can induce the production of short-chain fatty acids (SCFAs), including acetic acid, propionic acid, and butyric acid. Recent studies have shown that propionate downregulates FA levels in serum and reduces food intake [62]. In the small intestine, the PPARα agonist Wy-14643 regulates the expression of proteins involved in FAO and ketogenic effects, such as carnitine palmitoyltransferase 1 (CPT1), to regulate cholesterol and glucose transport [100]. PPARα is involved in the regulation of transporters and related metabolic genes in the small intestine during fasting [101]. A HFD can cause microbial and physiological system disorders in the small intestine through the PPARγ pathway [71]. Pioglitazone can inhibit visceral allodynia and increase colonic permeability, but the PPARγ antagonist GW9662 completely reverses the effect of pioglitazone, indicating that pioglitazone may be used for irritable bowel syndrome (IBS) treatment [102] by activating PPARγ. In our previous research, DBZ can increase the production of beneficial bacteria, such as *Akkermansia*, while inhibiting harmful bacteria, such as *Helicobacter marmotae*, *Odoribacter*, and *Anaerotruncus* in diabetic mice sustained on a HFD, thereby restoring intestinal homeostasis and improving mouse body weight and insulin resistance [55]. As is well known, the treatment of the microbiome plays a certain role in the treatment of T2DM, NAFLD, and other diseases [103,104,105]. Although the current research results and clinical trials still cannot provide an effective treatment scheme, it is still essential to maintain energy metabolism of the microbiome by regulating PPARs, which is a potential target for future drug development.

### 3.4. PPARs in Skeletal Muscle

The largest metabolic human organ, the skeletal muscle, is mainly involved in the metabolism of the organism by maintaining energy homeostasis. In skeletal muscle, PPARβ/δ is mainly involved in lipid metabolism involving fatty acid oxidation and utilization. During exercise, PPARβ/δ plays an important role in regulating mitochondria in skeletal muscle. Knockdown of PPARβ/δ results in a decrease in PGC-1α and mitochondrial protein levels. Interestingly, leucine promotes mitochondrial biogenesis and oxidative metabolism through the PPARβ/δ pathway to increase GLUT4 levels and glucose uptake in myotubes [106]. Thus, PPARβ/δ is important for energy maintenance during exercise and adaptation to increased mitochondrial enzymes in skeletal muscle [107]. One of the other two subtypes, PPARγ, is involved in glucose uptake to induce insulin-stimulated glucose metabolism in skeletal muscle, and the other, PPARα, is related to muscle fibers. When PPARα is overexpressed, it induces a change in the type of muscle fiber that protects mice from diet-induced obesity. However, in PPARα-knockout mice, although oxidative muscle fibers are increased, fatty acid oxidation is reduced during starvation [41]. It has been reported that fasting results in a decrease in the rhythm of brain and muscle ARNT-like 1 (BMAL1) and REV-ERBα (NR1D1) in skeletal muscle and the regulation of transcription factors such as glucocorticoid receptor (GR), forkhead box protein O (FOXO), and PPARs to induce a switch in rhythmic gene expression [108]. Some studies have reported that PPARs regulate skeletal muscle metabolism through the biological clock or epigenetics, but further studies are still needed to lay a foundation for PPARs as future targets for the treatment of muscle diseases.

### 3.5. PPARs in the Pancreas

The pancreas is one of the most important organs in the mammal body and has the function of digesting lipids, proteins, and sugars. Since it has an exocrine function, physiological and pathological changes in the pancreas are closely related to energy metabolism. PPARβ/δ is widely expressed in rat and human pancreatic tissues. PPARβ/δ is abundantly expressed in pancreatic β-cells, but the expression levels of PPARα and PPARγ are relatively low [109,110]. In β-cells, PPARβ/δ is involved in mitochondrial energy metabolism and insulin secretion [109] and regulates the expression of genes associated with fatty acid expression. Activation of PPARβ/δ can reduce blood glucose levels and improve insulin sensitivity in db/db mice [111]. PPARγ improves fatty acid-related physiological responses in pancreatic β-cells [110]. Deletion of PPARγ leads to the disturbance of islet glucose metabolism, suggesting that it plays an important role in maintaining glucose metabolism [110]. Selenium can antagonize apoptosis induced by cadmium through the PPARγ/PI3K/Akt pathway in pancreatic cells in chickens [112]. In recent studies, PPARα was expressed in INS-1 cells to induce lipid accumulation as well as increase β-oxidation. Clearly, the role of PPARs in the pancreas has not been clearly elucidated; therefore, further research is needed to lay a foundation for the prevention and treatment of pancreatic cancer and other diseases in this organ.

### 3.6. PPARs in the Heart

PPARα is highly expressed in cardiomyocytes. It has been proved that PPARα deficiency would cause cardiac dysfunction, which was associated with structural abnormalities in mitochondria and downregulation of the cardiac antioxidant capacity. In a mouse model of heart failure, the expression of the cardiac PPARα target genes such as CPT-1 and fatty acid transporter 1 (FATP1) was also significantly reduced [113]. When PPARα is specifically activated by the agonist WY-14643, it can significantly improve cardiac function and attenuate cardiac fibrosis in mice [114]. Overexpression of PPARβ/δ in mouse hearts enhances mitochondrial synthesis and metabolism and myocardial oxidative metabolism, improving cardiac function, and reduces myocardial fibrosis [115]. In rats with congestive heart failure, the PPARβ/δ-specific agonist GW610742 significantly inhibited right ventricular hypertrophy and reduced the level of natriuretic peptide. The heart-specific knockout of PPARγ in mice leads to cardiac hypertrophy, which affects the metabolism and function of the heart [116]. Although there have been some studies on the role of the PPARs in the heart, the specific mechanism of regulating cardiac energy metabolism needs to be explored with the aim of providing potential targets for drugs developed to treat cardiovascular diseases.

## 4. Functions of PPARs beyond Being “Nutrient and Energy Metabolite Receptors”

The development of many diseases is associated with metabolic disorders, and PPAR agonists and antagonists are already used in treatments of glycolipid metabolism disorders, such as hyperglycemia and hyperlipidemia. In addition, increasing studies have revealed that PPARs also play an important role in many other diseases, such as inflammation, neuron disease, and cancer.

Inflammation is usually among the main causes of many kinds of diseases and is generally exacerbated in the development of disease. In inflammatory processes, PPARs generally have anti-inflammatory effects and three distinct means of involvement. First, PPARs and NF-κB compete with each other to bind the same set of coactivators [117]. The activity of PPARs is inhibited under normal conditions because of coactivator binding to nuclear receptor corepressors (NCoR). When corepressors are separated from their receptors, coactivators bind to them, and PPARs are activated to regulate inflammation. Through another mechanism, PPARs interact directly with transcription factors to inhibit inflammation [117]. For example, in endothelial cell lines, PPARα inhibits the inflammatory response by interacting with p65 directly [118]. Similarly, PPARγ inhibits the secretion of cytokines in LPS-stimulated macrophages by interacting with p65/p50 directly [119]. In addition to the two mechanism models described, PPARs can also inhibit NF-κB activation by enhancing the stability of corepressor complexes of NF-κB [120].

PPARs are also expressed in the central nervous system (CNS), including neurons and astrocytes, suggesting that PPARs may play important roles in neurodegenerative diseases. However, in the past there has been little research focus on this field. However, an increasing number of recent studies have shown that PPARs have many beneficial effects that prevent mitochondrial dysfunction, proteasome dysfunction, oxidative stress, and downregulation of neuroinflammation, which are the main causes of neurodegenerative diseases. Parkinson’s disease (PD) is a progressive and chronic neurodegenerative disease in which the pathological process involves progressive loss of dopamine neurons in the substantia nigra. The possible cause of the degradation of dopaminergic neurons includes oxidative damage, neuroinflammation, and mitochondrial dysfunction [121]. Some studies have shown that pioglitazone, a PPARγ agonist, protects the bodies of dopaminergic neurons in the substantia nigra and striatum from the effects of mitochondrial neurotoxin, such as 1-methyl-4-phenyl-1,2,3,6-tetrahydrodropyridine (MPTP) in mice [122]. Oral administration of pioglitazone could prevent MPTP-induced neurodegeneration and the loss of dopaminergic cells in the substantia nigra [123]. In addition, the effect of PPARγ in the well-known neuron disease Alzheimer’s disease (AD) has also been widely investigated. There is a pathophysiological link between T2DM and AD, including insulin resistance and reduced insulin secretion from pancreatic β-cells [124]. It has been reported that the risk of AD is decreased by 55% in patient populations treated with nonsteroidal anti-inflammatory drugs (NSAIDs). The mechanism for this treatment involved PPARγ activation due to the NSAIDs binding to it, inhibiting microglial activation and inducing the expression of a series of proinflammatory molecules [125]. In addition, PPARγ agonists reduced the activation of amyloid beta (Aβ) in microglia and prevented the death of hippocampus and cortical neurons through the anti-inflammatory effects of PPARγ [126,127]. One clinical study showed that people receiving 4 mg of rosiglitazone daily had improved memory and selective attention [128]. In addition, patients with T2DM and AD treated with pioglitazone also displayed cognitive and metabolic enhancements [129]. Therefore, PPARs are a potential therapeutic target for neurodegenerative diseases that should be further investigated.

PPARs also have important regulatory roles in some types of cancer. PPARα, PPARβ/δ, and PPARγ have different effects on the development of cancer. For colorectal cancer, PPARα activated by Wy-14643, a potent exogenous PPARα ligand, inhibits inflammation by reducing the levels of inflammatory factors to inhibit colorectal cancer [130]. PPARγ inhibits the development of colorectal cancer by regulating cell differentiation [131] and modulating the expression of cell cycle regulatory factors [132]. However, the role of PPARβ/δ in colorectal cancer still has no broad consensus. S Beyaz et al. demonstrated that diet-induced obesity increased the number and proliferative properties of intestinal stem cells (ISCs) by activating PPARβ/δ, which exacerbated intestinal dysplasia [75]. However, emergent studies have also found that the expression of PPARβ/δ is lower in colon tumor tissue compared to that in the corresponding normal tissue [133,134]. Furthermore, many laboratories have found that activation of PPARβ/δ is mediated by the APC-β-catenin-TCF4 pathway. This finding is not consistent with the results of many studies on human colon cancer [135]. For breast cancer, the PPARγ agonist GW7845 can inhibit *N*-nitroso-*N*-methylurea-induced breast cancer in rats [136] and treatment with troglitazone can also alleviate breast cancer induced by 7,12-dimethylbenz[a]anthracene (DMBA) in mice [137]. However, the role of PPARβ/δ in breast cancer remains controversial. Analyses of Human Protein Atlas data (http://www.proteinatlas.org/ENSG00000112033-PPARD/cancer) showed that the PPARβ/δ protein is increased in many types of human tumors. Furthermore, increasing the expression of PPARδ through a transgenic method enhances the migratory ability of cells in human breast cancer lines [138]. Some studies have shown that activation of PPARβ/δ by GW501516 significantly inhibits breast cancer cell migration and invasion by mediating thrombospondin-1 (TSP-1) and degrading protease [139]. Thus, there is still a long way to go to determine the exact effect of PPARβ/δ agonists in cancer development.

PPARs act as significant modulators in many diseases, hence, it is necessary to continue exploring the function of PPARs because their potential as therapeutic targets cannot be ignored.

## 5. Conclusions

Past studies of the PPARs identified in this review have illustrated the physical functions of these nuclear receptors. All these PPARs participate in the regulation of nutrient and energy metabolism in the organism and display a variety of roles in different tissues and metabolic pathways.

Generally, PPARα is the key master of lipid metabolism that is involved in regulating fatty acid transport, binding, lipogenesis, and oxidation through the mitochondria or peroxisomes and mainly in the liver [33]. PPARα improves the fatty acid influx from the plasma to the liver and then induces FAO in the liver, WAT, BAT, SKM, and intestine [34]. Another PPAR isoform, PPARγ, reduces the levels of fatty acids in circulation and improves fatty acid uptake, storage, and lipogenesis in WAT [25]. With adverse effects, the expansion of adipose tissue occurs mostly in the clinical application of TZD treatment [140]. The ability of PPARγ to regulate glucose homeostasis is based on multiple mechanisms. Similar to PPARα, PPARβ/δ facilitates the oxidation of fatty acids in the liver, WAT, and SKM, and it also lowers the levels of very-low-density lipoproteins (VLDLs) and TGs in plasma, but increases the HDL levels in serum [34]. In the regulation of glucose homeostasis, PPARβ/δ improves glycogen synthesis and decreases gluconeogenesis and the levels of glucose in blood by enhancing the utilization of glucose in SKM [46].

Due to the master regulatory roles of PPARs in lipid and glucose metabolism, their corresponding synthetic ligands have been applied in clinical therapies for T2DM, dyslipidemia, obesity, NAFLD, and other diseases [25]. While the agonists of PPARs have several weaknesses such as weakly potent PPARα agonists, adverse effects of PPARγ agonists, and no clear effects of PPARβ/δ agonists [25].

For example, increasing numbers of fibrate trials have addressed fenofibrate intervention and event lowering in diabetes (FIELD) and action to control cardiovascular risk in diabetes (ACCORD), and have revealed that activation of PPARα by fibrates has rarely reduced cardiovascular disease (CV) risk [141] and has also failed to meet the criteria for controlling chronic inflammatory diseases [142]. Perhaps the specific effects of fibrates require further investigation in future drug research.

TZDs have been used as first-line drugs for T2DM therapy in past decades, but are still largely controversial because of their adverse effects, which include weight gain, congestive heart failure, edema, and so on [143]. Hence, in the United States, it has been withdrawn from use (as pioglitazone has been) [144]. The unfavorable effect of PPARγ agonists involved in promoting proadipogenic activity has led to the targeting of these adverse effects as one of the solutions [25]. Interestingly, a recent study showed that inhibiting the activation of cyclin-dependent kinase 5 (CDK5) would decrease the levels of PPARγ phosphorylation, which is the cause of adverse effects [145]. This finding provides insight for drug research and for attenuating the adverse effect of PPARγ.

It is equally important to target PPARβ/δ for the management of multiple metabolic syndromes since it synergistically modulates lipid metabolism. Preclinical studies have revealed beneficial effects of PPARβ/δ in the management of metabolic syndromes such as obesity, T2DM and NAFLD, or NASH [146]. In addition, MBX-8025, an agonist of PPARβ/δ, showed a series of encouraging effects involving decreased levels of γ-glutamyl transpeptidase and alkaline phosphatase, which are indicators of primary biliary cholangitis [147]. As it elevates the risk of adenocarcinoma, the PPARβ/δ agonist GW501516 should be carefully observed in future preclinical studies [148].

Dual- and Pan-PPAR agonists:

Many compounds, mainly dual- or pan-PPAR agonists, have been tested in the past decade to lower the adverse effect of a single agonist to PPARs. For example, a sequence of pharmaceutical molecules showing the dual effects of PPARα/γ agonists, called “glitazars”, has been studied for T2DM therapy [149]. However, most of these drugs were discontinued after clinical trials due to the variety of adverse effects associated with them [143]. Saroglitazar, the only glitazar still being considered, has shown encouraging effects in the treatment of diabetic dyslipidemia and hypertriglyceridemia and is usually used for T2DM treatment combined with statins [150]. Elafibranor, a selective dual agonist of PPARα/β(δ), has beneficial effects by improving lipid and glucose homeostasis and increasing insulin sensitivity of peripheral and liver tissue in phase III trials [151,152]. Moreover, another finding demonstrated that elafibranor also has an effect in preventing NAFLD/NASH and liver fibrosis [153]. The dual agonist to PPARγ/β(δ), T3D-959, has been shown to enhance spatial learning and memory and thus prevents AD [154]. Furthermore, T3D-959 also improved motor abilities and protected both cortical and reverse white matter structure in a phase II a trial [155]. Similarly, increasing pan-PPAR agonists have been developed for disease treatment. Bezafibrate was the first pan-PPAR activator used in clinical practice. It has a good lipid-control ability and a good safety profile [156]. Although bezafibrate has a relatively low potency against PPARs [143], it still showed a lower incidence of atherosclerotic lesions and coronary events in a Bezafibrate Atherosclerosis Coronary Intervention Trial (BECAIT) [157]. A novel non-TZD pan-PPAR agonist, chiglitazar, has shown different AC50 values for three types of PPARs during phase III clinical trials in China [158]. Another pan-PPAR agonist, IVA337, displayed beneficial effects by preventing and reversing skin fibrosis and is currently in phase II clinical development for NASH treatment [159].

Selective PPAR modulators:

To ameliorate the adverse effects of PPARs and maximize the therapeutic potential of PPARs, several selective PPAR modulators (SPPARM) have been developed to enhance the pharmacological effects of PPAR agonists. Decreasing the affinity of PPAR agonists to cofactors or inducing their binding to different promoters of target genes may be mechanisms by which SPPARM reduces adverse effects [34,160]. One of the selective PPARα modulators, pemafibrate (K-877), has a higher PPARα-activated potency and greater selectivity for PPARγ [161]. In addition, pemafibrate decreased the levels of TGs and remnant lipoproteins in plasma and showed antiatherogenic profiles [162]. Even in phase III clinical trials, pemafibrate showed better activation of PPARα agonists and alleviated TG levels better than fenofibrate [163,164]. In our previous studies, DBZ, a potential PPARγ agonist, prevented diet-induced atherosclerotic plaques in ApoE^−/−^ mice by enhancing LXR activation and inhibition of inflammation [56]. DBZ also showed an anti-obesity effect and reversed microbiota dysbiosis in HFD-fed mice [55]. SA, a traditional Chinese herb, inhibits adipocyte differentiation partly by downregulating PPARγ and other genes involved in adipogenesis and glucose transportation [82]. In addition, seladelpar, a selective PPARβ/δ agonist, modulated the reversed cholesterol transporter ABCG5/ABCG8 during primary biliary cirrhosis treatment [58].

Collectively, the increasing number studies of PPARs have uncovered mechanisms of nutrient and energy metabolic homeostasis in different nutritional states. Revealing the roles of PPARs in various metabolic organs and pathological conditions will contribute to new therapeutic advances in treating many metabolic disorders.
